# Developing a dashboard to meet Competence Committee needs: a design-based research project

**DOI:** 10.36834/cmej.68903

**Published:** 2020-03-16

**Authors:** Brent Thoma, Venkat Bandi, Robert Carey, Debajyoti Mondal, Rob Woods, Lynsey Martin, Teresa Chan

**Affiliations:** 1 Department of Emergency Medicine, University of Saskatchewan, Saskatchewan, Canada; 2 Department of Computer Science, University of Saskatchewan, Saskatchewan, Canada; 3 Division of Emergency Medicine, Department of Medicine, McMaster University, Ontario, Canada; 4 McMaster program for Education Research, Innovation, and Theory (MERIT), Ontario, Canada

## Abstract

**Background:**

Competency-based programs are being adopted in medical education around the world. Competence Committees must visualize learner assessment data effectively to support their decision-making. Dashboards play an integral role in decision support systems in other fields. Design-based research allows the simultaneous development and study of educational environments.

**Methods:**

We utilized a design-based research process within the emergency medicine residency program at the University of Saskatchewan to identify the data, analytics, and visualizations needed by its Competence Committee, and developed a dashboard incorporating these elements. Narrative data were collected from two focus groups, five interviews, and the observation of two Competence Committee meetings. Data were qualitatively analyzed to develop a thematic framework outlining the needs of the Competence Committee and to inform the development of the dashboard.

**Results:**

The qualitative analysis identified four Competence Committee needs (Explore Workplace-Based Assessment Data, Explore Other Assessment Data, Understand the Data in Context, and Ensure the Security of the Data). These needs were described with narratives and represented through visualizations of the dashboard elements.

**Conclusions:**

This work addresses the practical challenges of supporting data-driven decision making by Competence Committees and will inform the development of dashboards for programs, institutions, and learner management systems.

## Abstract (fr)

**Contexte:**

Les programmes fondés sur la compétence sont adoptés dans la formation médicale à travers le monde. Les comités des compétences doivent visualiser efficacement les données d’évaluation des apprenants pour soutenir leurs prises de décision. Les tableaux de bord jouent un rôle essentiel dans les systèmes d’aide à la décision dans d’autres disciplines. La recherche orientée par la conception permet le développement et l’étude simultanés des environnements éducatifs.

**Méthodes:**

Nous avons utilisé un processus de recherche orienté par la conception au sein du programme de résidence en médecine d’urgence à l’Université de la Saskatchewan pour déterminer les données, les analyses et les visuels dont a besoin son comité des compétences, et avons développé un tableau de bord intégrant ces éléments. Les données narratives ont été recueillies auprès de deux groupes de discussion, lors de cinq entrevues et par l’observation de deux réunions du comité des compétences. Les données ont été analysées de manière qualitative pour élaborer un cadre thématique soulignant les besoins du comité des compétences et orienter le développement du tableau de bord.

**Résultats:**

L’analyse qualitative a dégagé quatre besoins du comité des compétences (explorer les données d’évaluation en milieu de travail, explorer d’autres données d’évaluation, comprendre les données dans leur contexte et s’assurer la sécurité des données). Ces besoins ont étédécrits avec des récits et représentés par des visuelsdes éléments du tableau de bord.

**Conclusions:**

Le présent travail aborde les difficultés pratiques de soutenir une prise de décision fondée sur des données par les comités des compétences et oriente le développement des tableaux de bord pour les programmes, les établissements et les systèmes de gestion des apprenants.

## Introduction

Competency-Based Medical Education (CBME) programs are being implemented in residency programs around the world. One of the core components of CBME is programmatic assessment.^2^ The Royal College of Physicians and Surgeons of Canada has committed to CBME through the Competence By Design (CBD) model.^3^ Within this model, programmatic assessment^4^^,^^5^ requires faculty to observe residents’ work^6^ and provide them with frequent, low-stakes assessment of entrustable professional activities (EPAs) using a five-point entrustment score.^7^^-^^9^ Competence Committees (CCs) review these data on a regular basis to provide the residents with feedback on their performance and determine when they have demonstrated the competence required to progress to the next stage of training or enter practice.^10^^,^^11^ However, the transition to CBME is resulting in volumes of assessment data that dwarf those seen in traditional assessment programs.^12^ CCs are struggling to make robust, data-driven decisions while also providing feedback that fosters resident development.^11^^-^^16^

To realize the promise of CBME, resident assessment data need to be organized and displayed effectively^17^^,^^18^ Analytical and visualization techniques have been developed in other fields (e.g. sport and business) to address the challenges presented by large datasets.^12^ The subfield of learning analytics uses large datasets, statistical techniques, and predictive modeling^19^ to describe, characterize, and predict the learning behaviour of individuals^20^^,^^21^ Dynamic dashboards, described as “a visual display of the most important information needed to achieve one or more objectives” are frequently used to consolidate and arrange these dataso the information can be monitored at a glance”.^17^^,^^22^The development of such dashboards is an iterative process^18^ and requires collaboration with information technology experts, assessment experts, data managers, and dashboard users.^23^

Within the context of the University of Saskatchewan emergency medicine residency program, we sought to identify CC needs and design a dashboard containing elements (data, analyses, and visualizations) that meet their needs.

## Methods

We utilized a design-based research approach^24^^-^^26^ and followed best practices^23^^,^^26^^,^^27^ to meet this objective. We report the qualitative components of our project in keeping with the consolidated criteria for reporting qualitative research.^28^ The University of Saskatchewan Research Ethics Board (BEH ID 463) deemed our research project exempt from ethical review.

### Setting and participants

Our project was situated within the Royal College of Physicians and Surgeons of Canada Emergency Medicine residency program at the University of Saskatchewan during the 2018-19 academic year. During the period of study, the residency program had 14 residents enrolled from post-graduate years one to five. Beginning on July 1, 2018, all residents in the program were assessed using the emergency medicine CBD EPAs.

The members of the program’s CC during the 2018-19 academic year were the subjects of the research. Our CC was created on July 1^st^, 2017 and evolved for a year prior to the onset of this project. All CC policies and procedures were formally adopted by the Emergency Medicine Residency Program Committee before the start of the research project. The CC contained five members including the Program Director (RW), CC Chair (LM), two emergency medicine faculty members, and a non-physician healthcare professional. Scheduled meetings were held quarterly (September, December, March, and June) over a three-hour period with additional meetings convened on an *ad hoc* basis by the CC Chair. The CC reviewed the assessments of every resident at each of the quarterly meetings.

### Research team

We assembled a diverse team of collaborators to conduct our study including an established medical education researcher (BT), longstanding program director (RW), CC chair (LM), external expert in medical education research and assessment (TMC), emergency medicine resident (RC), computer science professor (DM), and computer science Master’s student (VK).

### Design-based research process

We employed a design-based research methodology.^24^^-^^26^ Design-based research is an “authentic, contextually aware, collaborative, theoretically focused, methodologically diverse, practical, iterative, and operation-oriented” process^24^^,^^29^ which aims to bridge research and practice in education by integrating investigation and intervention.^24^^,^^26^^,^^30^ The research process followed the four phases of design-based research^24^^,^^26^ with the dual objectives of investigating the needs of CC members and creating a dashboard which meets these needs.

### Phase 1. Analysis and exploration

BT reviewed the literature on CC function,^11^^,^^16^^,^^31^^-^^33^learning analytics,^12^^,^^21^ and data visualization.^18^^,^^22^^,^^23^^,^^27^^,^^34^ He summarized this work for our team’s programming experts (DJ and VK) to provide the context required to support dashboard programming. In September 2018, BT took field notes at the first CC meeting of the academic year and facilitated an 83 minute, in-person focus group with our local educational (RW and LM) and programming experts. The primary questions guiding the field notes and asked of the focus group were: What data are required for the CC to make resident assessment decisions? How should these data be presented?

### Phase 2. Design and construction

The narrative data from Phase 1 was transcribed and qualitatively analyzed to inform the development of the initial dashboard. VK and BT met two-to-four times monthly to design the prototype dashboard, which incorporated the elements required to support CC decision making. The first CC dashboard prototype was used at the CC’s second meeting in December 2018. Phases 2 and 3 alternated throughout the remainder of the academic year with each data collection and analysis spurring dashboard design changes.

### Phase 3. Evaluation and reflection

CC members evaluated and reflected on the dashboard on two additional occasions during the academic year. In March 2019, BT took field notes at the third CC meeting of the academic year and facilitated a 32-minute, in-person focus group with the local educational experts (RW and LM). In June 2019, BT conducted phone-based interviews with all five members of the CC that ranged in length from 22 to 46 minutes. During the interviews, the CC members were asked to talk through their use of the dashboard with emphasis on what they did or did not find useful and what they would like to have added or modified. Following each of these events, the narrative data was transcribed and analyzed to inform the design and construction of the dashboard (Phase 2).

### Phase 4. Implementation and spread

The implementation and spread of the dashboard is ongoing. Locally, we presented the dashboard at our institution’s postgraduate medical education committee to demonstrate how it is being used by our CC. This spurred interest from other programs, and it has now been adapted for use by the pathology, obstetrics and gynecology, neurosurgery, and internal medicine programs at the University of Saskatchewan. We anticipate that additional local residency programs will begin using the dashboard soon. It is also being adapted for use by our undergraduate program to support the Association of Faculties of Medicine of Canada’s EPA project.^35^

External inquiries regarding the dashboard and the research process were received from the Royal College of Physicians and Surgeons, the University of Calgary, the University of Ottawa, the University of Manitoba, and the Elentra Consortium.^36^ The University of Calgary has formally endorsed the use of a simplified version of the CC dashboard (https://cbme.usask.ca/#/Tools) for the visualization of their residency programs’ CBD data. BT met with individuals and/or groups from each of the external organizations, described the dashboard and the design-based research process, and received further feedback that informed minor changes to the design of the dashboard. This feedback could not be formally integrated into the qualitative analysis as it was outside of the scope of the research ethics application.

### Qualitative analysis

The narrative data originating from the field notes, focus groups, and interviews were analyzed to identify the CC’s core needs and the dashboard elements (data, analytics, and visualizations) required to meet them. Comments that were not germane to the topic of interest - the needs of the CC - were excluded from the analysis. Excluded comments focused on faculty development and program evaluation and will be analyzed and reported in subsequent manuscripts along with dashboards designed for these purposes.

The qualitative analysis was conducted using a constructivist grounded theory approach and constant comparative method.^37^ BT developed a preliminary codebook that was populated with representative quotes for each code as the data was collected. BT also compiled the codes into a preliminary framework outlining CC member needs and dashboard elements that addressed them. The codebook and framework were revised as additional data were collected.

To ensure the rigour of the analysis, a second investigator (TMC) reviewed all transcripts and contributed to the development of the codebook and framework on a delayed timeline. The use of a single primary reviewer with delayed secondary review was a pragmatic decision made to expedite the interpretation process by reducing analysis delays that would slow the dashboard design process. Consensus between the two coding investigators (BT and TMC) was reached through discussion on all changes. Following the completion of the qualitative analysis, representative quotes and images demonstrating the data and its analysis or visualization were selected to characterize each theme. The data collection phase in which the participants described or suggested modifications to each of the elements was tracked.

Throughout the coding process, the investigators considered their own positionality and its potential impact on their interpretation of the data. BT is an emergency physician with advanced training in educational research who has been involved with the residency program as a Program Director, CBD Lead, and CC Chair. He is currently a Residency Program Committee member. TMC is an emergency physician with advanced training in educational research and qualitative methods. She is also the current CC Chair for the McMaster Emergency Medicine Residency Program and has created a learning analytics and data visualization dashboard for her program.^38^^-^^41^ She acted as an external collaborator, providing a literature- and experience-informed perspective to the analysis.

Participant checks with the CC members occurred in two ways. First, throughout the development process the CC utilized the developing dashboard and suggested changes if/when the dashboard elements were not consistent with their needs. Second, each of the CC members was sent a copy of the final thematic analysis and asked to comment on any aspects that were not in keeping with their perspective.

### Data management and dashboard programming

Throughout the study, all EPA assessment data was entered by Faculty into the Royal College of Physicians and Surgeons MainportePortfolio (Ottawa, ON). This data was exported and uploaded into our dashboard each Monday by our Program Administrator. During the upload process the EPA data was reformatted and tagged with additional data from the residents’ profiles including the rotation and stage they were in when each EPA was completed. Contextual and non-EPA information (e.g. resident name, program start date, phase of training, rotation schedule, exam scores) was entered in the dashboard by our Program Administrator. All dashboard data was stored on a secure server within the Department of Computer Science at the University of Saskatchewan.

The dashboard was developed on a distributed web architecture consisting of three main parts: A web server for hosting the website, a database server to securely hold the data, and a backend server to authenticate users and perform CRUD (create, read, update, and delete) operations on the database. This distributed structure allowed each component to be coded independently. This was essential because the project required rapid prototyping in response to CC member feedback. Additionally, this allowed the dashboard to be scaled easily as additional residency programs began to use it.

Functionally, the dashboard website creates its visualizations in real time using data served by the backend server. The visuals are rendered in a Scalable Vector Graphics (SVG) format that is both scale and transform invariant. This makes the CC member experience interactive and consistent across various screen sizes and orientations. Data security was ensured by authenticating users using the University of Saskatchewan’s Central Authentication Service. Access to data was restricted based on preassigned user roles (Resident, CC Member, Academic Adviser, Program Director, and Program Administrator). To facilitate the rapid dissemination and replication of the dashboard, we publish its up-to-date code under an open access license on Github.^42^

## Results

The qualitative analysis identified four CC member needs and seventeen potential dashboard elements (Table 2. See Appendix A). During the participant check this description of CC needs was reviewed and endorsed by all CC members without suggestions for additional changes. Due to the limitations of tables and figures, we produced a video walk-through of the dashboard outlining the representation of each of the elements (Video 1 – available at https://youtu.be/l8n6s-y3mko).

While the four primary needs were mentioned in each of the study’s phases, in some cases the CC members’ perspectives evolved over time. For example, initially the CC members requested normative comparisons of each resident’s EPA metrics. However, over time their focus shifted towards contextualizing each resident’s EPA metrics using the rotations they had recently completed and their stage of training. Over time, additional needs were also identified. Table 1 outlines the first time that each of the CC needs and dashboard elements were described (mentioned in bold) or modified.

**Table 1:** Outline of the dashboard elements requested during each of the three data collection periods.

Design and Construction (September 2018)	Evaluation and Reflection 1 (March 2019)	Evaluation and Reflection 2 (June 2019)
*1. Explore Workplace-Based Assessment Data*		
**1.1 EPA Acquisition Metrics** **1.1.1 Comparative EPA Metrics** **1.1.3 Expired EPAs** **1.2 Quantitative EPA Data** **1.3 Narrative EPA Data** **1.4 Narrative Assessments**	1.1.1 Comparative EPA Metrics **1.1.2 Contextualized EPA Metrics** 1.2 Quantitative EPA Data **1.2.1 Clinical Presentation and Patient Demographics**	1.1.2 Contextualized EPA Metrics
*2. Explore Other Assessment Data*		
**2.1 Resident Self-Assessment** **2.2 Competence Committee Decisions** **2.4 List of curricular requirements**	2.1 Resident Self-Assessment **2.3 Exam Scores**	
*3. Understand the Data in Context*		
**3.1 Efficiency** **3.2 Rotation Schedule** **3.3 Date Filter** **3.4 Rater Context**	3.1 Efficiency 3.2 Rotation Schedule	3.1 Efficiency **3.1.1 Orienting Features**
*4. Ensure the Security of the Data*		
**Legend:** -Dashboard elements being described for the first time within one of the three data collection periods are listed in bold text. -Dashboard elements which had modifications suggested within a data collection period are listed in standard text. -Dashboard elements for which there were no suggested changes within a data collection period are not listed.		

### 1. Explore workplace-based assessment data

**Figure 1:**
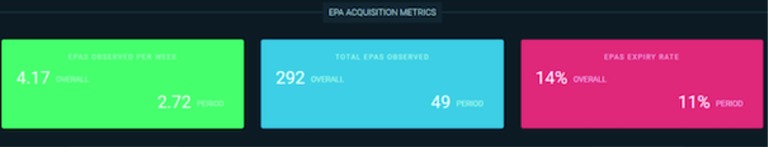
Visual representation of the EPA acquisition metrics displayed since the beginning of the resident’s participation in the competency-based assessment program and for a selected period.

A primary need of the CC members was to know if the residents were acquiring EPAs at an appropriate pace overall and since the last CC meeting. We developed numerical EPA acquisition metrics that were displayed near the top of the resident profiles. They included the number of EPAs observed per week, number of EPAs observed, and EPAs expired (Figure 1). These metrics were calculated since the beginning of the assessment program and within a customizable date range that was often used to isolate the period since the last CC meeting.

**Figure 2:**
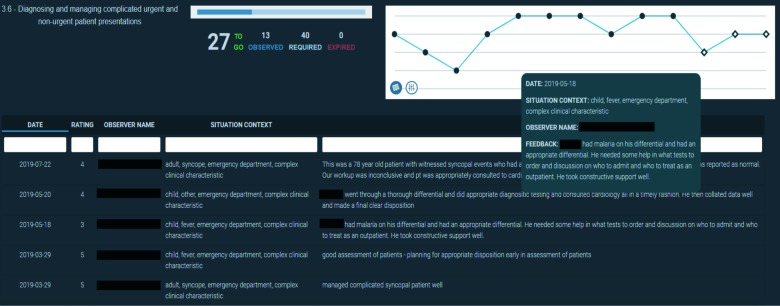
Visual representation of the achievement of a single entrustable professional activity assessments incorporating numerical metrics, a graphical representation of entrustment scores over time, and narrative feedback.

The CC members requested that the numerical entrustment scores for each EPA be represented in a graphical format that allowed the visualization of trends. They also wanted to know how many assessments of each EPA were needed and how many had expired. EPA-specific visualizations (Figure 2) provide CC members with the name of the EPA, the residents’ progress in receiving assessments (the blue progress bar and number of assessments required, expired, observed, and remaining), and a graphical representation of the entrustment score received on each assessment (bottom row = “I had to do”; top row = “I didn’t need to be there”).^8^^,^^9^

After viewing the numerical entrustment scores, the CC members needed to review the context and narrative comments for each EPA. We facilitated this in two ways that were both found to be useful (Figure 2). Hovering over an individual data point displays its associated narrative comment along with its context variables. This was useful when a CC member wanted to review specific assessments. Similar data can be displayed in a searchable and sortable tabular format. CC members found this table useful when they wanted to review all the feedback on an individual EPA.

**Figure 3:**
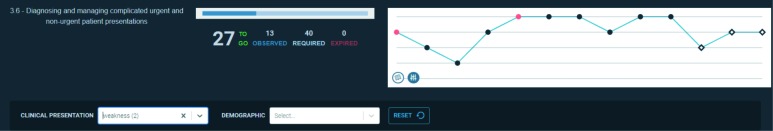
Visual representation of the achievement of entrustable professional activity assessments highlighting specific clinical presentations and/or patient demographics.

Some of the EPAs in the emergency medicine assessment program require the observation of specific clinical presentations and/or patient demographics. The CC members needed to determine whether the EPA data they were reviewing was representative of these requirements. We developed a mechanism to highlight selected clinical presentations or patient demographic to meet this need (Figure 3).

**Figure 4:**
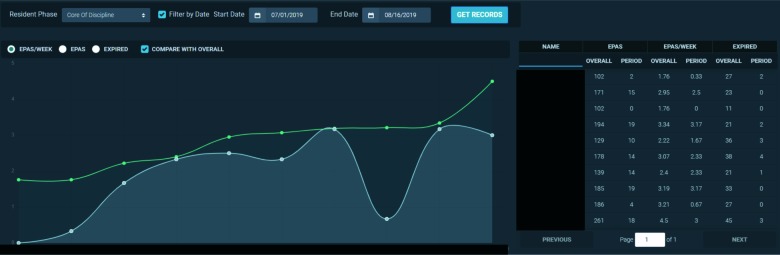
Visual representation of residents’ acquisition metrics plotting the number of overall entrustable professional activity assessments per week (y-axis) of each resident (x-axis) since the beginning of the resident’s participation in the competency-based assessment program (green line) and for a selected period (blue line).

Some CC members compared the progress of individual residents to each other to determine whether their acquisition of assessments was similar. We facilitated this by creating a normative visualization that compared the acquisition metrics of the residents (Figure 4). This visualization can display all residents or only those in a specific stage. Each metric can display data from the beginning of the assessment program alongside data filtered from a selected period.

**Figure 5:**
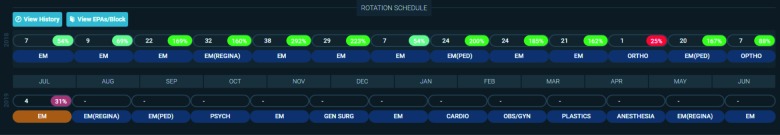
Visual representation of the number of entrustable professional activities observed for a single resident on each rotation with a heat map indicating the proportion of expected assessments (<25% of expected red; >80% expected green).

As alluded to previously, the CC members focus on normative data decreased throughout the year. Rather than comparing residents to each other, CC members began comparing their performance to the expected performance of residents on a given rotation. To support these comparisons, we developed a visualization of each residents’ rotation schedule that demonstrated the number of EPAs observed in each rotation relative to the number expected for a resident on that rotation as a percentage (Figure 5). The expected value for each rotation was determined by the Program Director after reviewing historic program evaluation data for each rotation. This percentage was heat mapped with values colored on a gradient from red (25% of expected or less) to green (80% of expected or greater).

**Figure 6:**
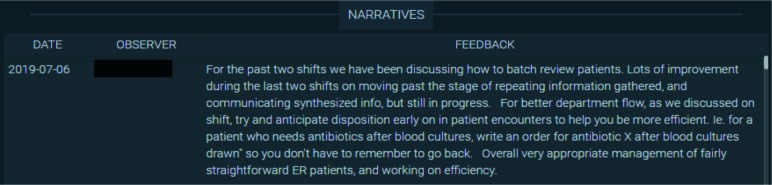
Tabular presentation of non-EPA narrative assessment data for an individual resident.

The CC also reviewed non-EPA narrative assessments. These assessments were not associated with an entrustment score and often related to either a resident’s overall function in the workplace or a particularly positive/negative assessment that did not fit into a specific EPA. These assessments do not include a numerical value, so we display them in a simple tabular format that is sortable by date and observer (Figure 6).

### 2. Explore other assessment data

**Figure 7:**
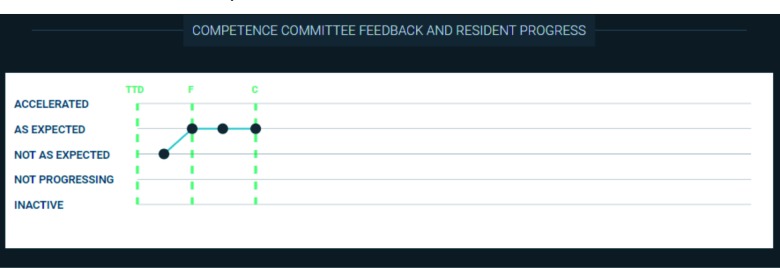
Visual representation of the status of a resident within their residency program over time incorporating narrative feedback from the Competence Committee.

**Figure 8:**
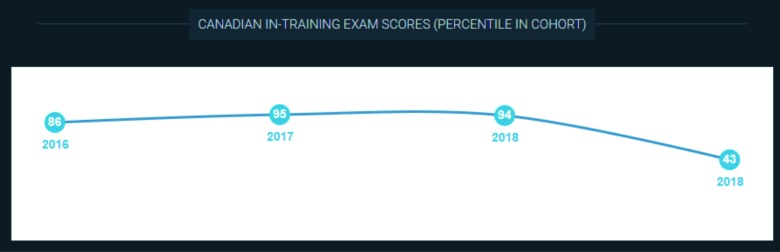
Visual representation of the within-cohort percentile rank score of an individual emergency medicine resident on their national written exam from 2016 through 2018.

**Figure 9:**
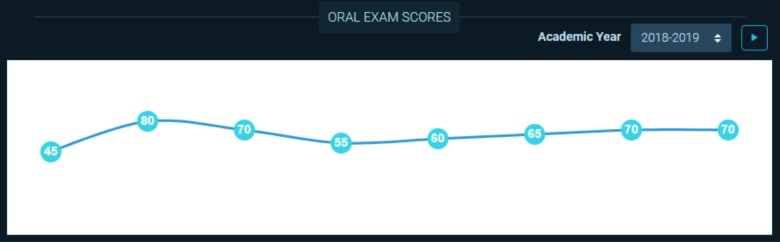
Visual representation of the oral examination scores of an individual resident in the 2018-19 academic year.

The CC members were also informed by other sources of assessment data including:

- Resident self-assessments: These assessments were completed quarterly prior to each CC meeting using a standard template (Appendix B). They provided CC members with insight into how the resident believed that they were progressing. The self-assessment form evolved over the year, with some changes related to the incorporation of additional information within the dashboard.
- Narrative feedback from previous CC decisions (Figure 7): CC members wanted to review each residents’ promotion status (e.g. ‘progress is accelerated’, ‘progressing as expected’, ‘not progressing as expected’, ‘failure to progress’, or ‘inactive’) over time. Hovering over each data point displays the narrative feedback that was provided to the resident by the CC. The green vertical lines indicate the initiation and completion of each stage of training.
- Resident performance on the emergency medicine Canadian In-Training Exams (Figure 8): The CC tracks these scores to assess the residents’ medical knowledge base and whether they are on track in their preparation for their national written examination.
- Resident performance on local oral examinations (Figure 9): The CC tracks these scores to assess each residents’ oral exam performance and preparedness for their national oral examination. Hovering over each point displays the context (e.g. the examiner and focus of each case) and the feedback the resident received from the examiner. Exams from the most recent year are displayed by default. Historical results can be loaded by selecting a prior academic year.
- Curricular requirements: Beyond their rotations, our residents complete numerous other tasks in each academic year (e.g. courses, shadow shifts with allied health professionals, an x-ray module, presentations at designated rounds, etc). A list of each residents’ curricular requirements was identified as a need as it allowed the CC to determine whether the residents were staying on top of these tasks. However, given the frequent modifications to the list and its specificity to a single residency program, it has not yet been incorporated into the dashboard.

### 3. Understand the data in context

Prior to the first CC meeting, the Chair spent substantial time organizing resident assessment data. The CC members needed a system to present the data in an intuitive, contextualized, fast, and accessible way. We designed the dashboard to present data from broad (top) to specific (bottom). We aimed to reduce the number of clicks required to review any single piece of data to a minimum. We reduced loading times to milliseconds by utilizing client-side data processing.

One CC member requested additional orienting features, as they did not find the minimalist presentation to be intuitive. Suggestions were made to include additional labels and an orientation guide that were felt to be especially helpful for new CC members.

As the number and type of EPAs completed by a resident during a given period varied with their rotation, CC members frequently referenced residents’ rotation schedules. An up-to-date rotation schedule (Figure 5) was incorporated prominently at the top of the dashboard to provide this orienting information.

The CC members wanted the EPA data to be reviewed at each meeting to be easily identifiable. To facilitate this, we created a date filter that changes the shape of each data point within the selected date range. As seen in Figures 2 and 3, the data points within the selected date range are displayed as open diamonds instead of black dots. This allowed the CC member to review only the relevant EPAs while preserving the perspective provided by seeing overall trends.

Some CC members alluded to variability between Faculty raters in terms of both the quality of their feedback and their credibility. Beyond making it easy to see which faculty member completed each assessment, we were unable to incorporate guidance on feedback quality or faculty credibility into the dashboard because methods to quantify assessor credibility and feedback quality within EPA assessments have not been developed or validated.

### 4. Ensure the security of the data

The CC members felt security of the assessment data was important given its sensitivity. They believed that compromised data could be used inappropriately to inform hiring, licensing, or medico legal decisions. In contrast, the CC members required easy access to this information for their work. Balancing these concerns, the dashboard was made accessible to all CC members through an online portal utilizing their standard University of Saskatchewan login credentials. This access was sufficiently convenient that CC members no longer shared spreadsheet exports containing resident assessment data. This likely increased security as CC members stored less resident data within their email accounts and/or personal computers.

## Discussion

We described a design-based research project that both developed a framework outlining the needs of CC members for various dashboard elements (data, analytics, and visualizations) and created a dashboard containing those elements.

Previously, authors have hypothesized that design- or action-research based frameworks like Design Thinking may hold the key to improving medical education,^26^^,^^43^ but this represents one of the first reports to harness the power of collaborative co-design to support the decision-making processes of CCs. Previous literature on CCs has focused largely on how they make their decisions^11^^,^^16^^,^^33^ with studies on pediatrics residents determining the weight given to various types of data (e.g. rotation ratings, faculty comments, personal experience with residents)^31^ and investigating how CC members identified residents with performance concerns.^32^ We believe that this work is complemented by our own, which pragmatically focused on determining what information CCs need and how it can be provided effectively. Further, our work has contributed to the literature by providing a thematic framework outlining CC needs.

The strengths of our approach include the detailed description of our research process and the visual presentations of its results using text, figures, and video. We believe that this outline will provide an accessible roadmap for CCs struggling to utilize their assessment data effectively. Further, the resulting dashboard has been published under an open access license to ensure that anyone with the requisite technical expertise and an assessment system based upon EPAs is able to adopt it.^42^

Interestingly, the CC members’ needs changed throughout the research process. As visualized in Table 2, new ideas for elements and modifications of old elements continued to arise over time. This was best exemplified within elements for comparative (1.1.1) and contextual (1.1.2) EPA metrics. The underlying CC member need for these metrics was an understanding of a resident’s over- or under-performance. At the beginning of the year there was a focus on quantifying performance in terms of the acquisition of EPA assessments between residents. However, as the year went on there was an increasing focus on understanding a resident’s performance (e.g. number of EPA assessments) within the context of their expected performance. This shift is best exemplified through the visualizations outlined in Figure 4 (which compares the resident’s performance to the other residents) and Figure 5 (which compares the resident’s performance to the Program Director’s expectations for a given rotation). It is unclear to what degree this evolution occurred due to the development of the CC (33) versus the availability of the dashboard, however, it is likely that both played a role. Of note, there was little discussion of the guidelines for EPA acquisition provided by Royal College, although they were incorporated into some elements (Figures 2 and 3). When available, it is likely that national CBME data will impact our CC’s interpretations by providing a broader perspective on resident achievement.

### Future directions

Dashboards provide both solutions and challengesfor CCs. Our study found that the needs of CC members can evolve over time, so ongoing revision of the dashboard will likely be required. CC members must also be aware that, while they have access to substantial amounts of data to support their decisions, they are still subject to their own biases.^41^Recent work suggests that there are multiple perspectives on how best to interpret portfolio data^44^ and further investigation will be required to determine how data, analytics, and visualizations impact CC decisions. In keeping with this, dashboard developers must also consider how our own perspectives and biases may be perpetuated within the design of a CC dashboard.

Moving beyond CCs, we plan to utilize a similar design-based research process to design dashboards that support resident learning, faculty development, and program evaluation in competency-based training programs.

### Limitations

This work has several limitations. First, it was not the goal of this research to evaluate the impact of the dashboard on CC function. While this would be an important finding that would support the effectiveness of our process, it goes beyond the scope of our current study’s objectives. Second, the generalizability of our results may be limited due to it being situated within a single emergency medicine residency program. However, it is notable that the competency-based assessment system follows the national framework for CBD^3^ and our findings are therefore likely to be relevant nationally both in emergency medicine and other specialties. Notably, the fourother specialties that have begun using the dashboard locally have endorsed its utility beyond emergency medicine. Third, the iterative design-based research process allowed CC members to utilize the dashboard as it was built which generated additional insights but required constant modifications and additions. While additional dashboard iterations could have been incorporated, we are confident that our current thematic framework is representative of our CC members needs given that only a small number of minor suggestions for new features and/or modifications occurred in the final data collection period. Lastly, the involvement of BT in the research process may have biased our findings. His familiarity with the residency program could be both an asset that helped to understand the context of the program and a liability that limited the potential for diverse interpretation of the data. We attempted to remediate this through the inclusion of an external investigator (TMC) in the analysis process.

### Conclusion

This project addresses the practical challenges of presenting assessment data to CCs. We anticipate that both the thematic framework and the dashboard elements that we developed will inform the development of CC dashboards for other CCs, institutions, and learner management systems. Design-based research could be used by others to support the design and study of educational dashboards.

## Appendix A

**Table 2:** Thematic analysis of Competence Committee needs, associated dashboard elements, and representative quotes.

CC Member Needs	Dashboard Element	Quotes
**1. Explore Workplace-Based Assessment Data**		FG1: A big part is specific EPA data. So, from each of the numerical EPAs. And then the narrative comments that go along with those. I4: First I look at all the outliers, if any of the EPA ratings that are 3 or less. And then go through the actual feedback with those just to see if they’re actually correlated. If the feedback correlates with an EPA rating that they got.
	**1.1 EPA Acquisition Metrics (Figure 1)**	I2: So usually would start just by looking at kinda total number of EPAs observed. So the EPAs per week total and then the expired ones that they have. And then I would just break those down based on the numbers for the last EPA period just to have an overall idea of how the resident has done.
	**1.1.1 Comparative EPA Metrics (Figure 4)**	FG2: Just kinda overall within residents within specific stages would kinda compare total number of EPAs with different rotations done just to see kinda what the trends were for residents in different years. I2: I think we’re happy – or I’m at least happy – without the comparison data on just looking at the resident metric dashboard just because we wanna get out of that mindset of potentially comparing residents. ‘Cause it may put undue pressure on certain residents.
	**1.1.2 Contextualized EPA Metrics (Figure 5)**	FG1: I think currently where it’s most helpful is seeing how many EPAs residents are getting on specific rotations. Because if you have one resident that rotates through general surgery and they’re getting 15 EPAs and then another one that comes through and they’re only getting three to five, then that kinda helps kind of assess them from that perspective as well. I5: … then you could see that their numbers were also low, then it would be a flag to talk to the resident to see what’s going on… To take it another level, it’d be nice to see like a target for each rotation in terms of what the history was last year.
	**1.1.3 Expired EPAs (Figure 1-3)**	I3: Sometimes the attending just doesn’t fill it out. Like they can’t get any more. But to get an idea of how many are expiring in general and in particular for that resident
	**1.2 Quantitative EPA Data (Figure 1-3)**	I2: The trend is the most important thing. So it’s looking at the overall number and then what they’ve done ‘cause you can clearly see the trend if they’re down in the 2s and 3s versus if they’re up in the 4s and 5s.
	**1.2.1 Clinical Presentation and Patient Demographics (Figure 3)**	I1: Or did they present to you sort of a representative sampling of procedures that they would be expected to do in the ED? I3: Just to understand where, like what they’ve been experiencing, where there may be gaps. Maybe they’re only getting like middle-aged people and they’re not getting the geriatric experience, for example.
	**1.3 Narrative EPA Data (Figure 2)**	I1: So it’s handy actually to have the narrative feedback where you can just sort of look with one click or one mouse over to see all of the things that have been said in that area. So that’s a big timesaver.
	**1.4 Narrative Assessments (Figure 6)**	I4: I just use (narrative assessments) to get an overall picture of how the resident’s doing. If they all sort of paint the same picture then it’s great and you get a better feel of where they’re at then just the feedback data on the EPA. They tend to be a little bit more in-depth. So it just gives you a better overall picture and a better – just gives you a better feel of where they’re at.
**2. Explore Other Assessment Data**		FG1: I suspect because so much of this information is hard to collate together, we probably haven’t even dreamed up what would even be the best. Because once we actually have some sort of usable interface to look at data, we can look at more of it and expect more of it. Whereas right now I think we’re just wrapping our heads around collating bits and pieces from so many sources […] But if it was all on one interface dashboard, we would look at it and go, “Awesome, this would be a great place to now add this bit and this bit and this bit.”
	**2.1 Resident Self-Assessment**	I4: [Resident self-assessments] are very useful, mostly ‘cause it kinda summarizes a lot of the data that you get from the EPAs. So it gives you a little bit more of a background of what they were on... then I get a bit of a better idea of where they think they’re going in the next few months. And it really helps me with their goals especially. So their goals for their past rotation, their goals for their next rotation. And that way you can kinda follow-up with their EPAs and correlate their EPAs with their goals that they identified and make sure that they’re actually getting to where they wanna be.
	**2.2 Competence Committee Decisions (Figure 7)**	I1: So I try to look through the report or the minutes from the previous competency committee to just refresh my memory on what we were saying our priorities for the resident were.
	**2.3 Exam Scores (Figure 8-9)**	FG2: So we do now twice annual written exams and once annual mock oral exams. And so it would be nice to see like a running tally of their exam scores across the years to see where they’re trending and where they rank.
	**2.4 List of curricular requirements**	CC2: What are they missing? Scholarly activities? Required activities for the year that have been ticked off - how many do they have checked off? Should be a constant reminder for them. A 'tick sheet' of their activities. I5: And then similarly… being able to tick off like I’ve done PALS, I’ve done my airway course, I’ve done my toxicology shifts. I’ve finished [the research course]. I’ve finished [the] Indigenous wellness [course]. I finished [the] ImageSim [modules]… so it’s very obvious when a resident hasn’t finished something.
**3. Understand the Data in Context**		FG2: I would look at EPA numbers just overall to get a sense of how many they’re doing. Then I would focus in on – I’d have a quick scan of what rotations they’d done recently to get a sense of whether or not that was a reasonable number of EPAs or not. Then I would move down into the specific stage of training that they were in and I would look at the EPAs they’d done in terms of scores as well as narrative comments. And I would filter it for the last three months to make sure I’m looking at the most recent data. […]And then I would take their narrative comments from previous – like their previous summary of how they were doing and what they wanted to work on to make sure that those things had been incorporated into this quarter of their work.
	**3.1 Organization for Efficiency**	FG1: It’s, yeah, from our perspective I think it’s more [the dashboard’s] organization and having things like readily available as opposed to the [previous] system right now where it’s clickclick download click click. So it’s more the things together that can be easily accessed.
	**3.1.1 Orienting Features**	I1: maybe just like a one-page job aid to how to get the most out of the dashboard. Just so that if there’s anything like that, people could quickly just scan a one-page summary and say, “Oh! I see it can do that. I didn’t realize,” or something like that. I4: I think one thing that would help people especially the new people coming in is just to have the icons – if you could label the icons. Then the little dropdown menus that you have, just to get an idea of what they actually are. Some of the things that you don’t realize you can – initially I didn’t realize that I could actually click on these so I was always kinda fumbling through this.
	**3.2 Rotation Schedule (Figure 5)**	FG1: It’d be nice also if you could have a bar of like what rotation – clinical rotation – they were on. So you could be like, “Oh well they didn’t get many this month but they were on plastic surgery, and we know that they’re only gonna get a handful.” But then the next block they were on emerg and then they got only seven which is way below what we’d expect.
	**3.3 Date Filter (Figure 2)**	I4: The first thing I do is I try and narrow down the data just from the period that we’re looking at. So I just try and get the date filter right for just the block that we’re looking at. And eliminate all the other pieces of the data to make it a little bit cleaner to look through.
	**3.4 Rater Context**	FG1: The quality of the data we have varies from faculty to faculty. Some are very good about filling out EPAs and getting a sense of what they mean. Other faculty don’t understand as much. There’s also quite a variability in the quality of the narrative comments. Some people are very descriptive and get to the heart of where the residents’ thought processes are. And other faculty write very non-descript vague statements about what was done.
**4. Ensure the Security of the Data**		FG1: It doesn’t matter to me where [the data is] stored as long as it’s secure. In terms of where it’s viewed, as long as we can – all committee members – can access it and look at changes to the screen in real time. I2: So I think it actually helps enhance security for what we’ve been doing for our resident reviews as opposed to like downloading of the Excel documents I5: the stakes of this data being compromised are much higher because you could have a PGY5 resident applying for a job somewhere and someone diving into their stuff. If they saw something they didn’t like might say, “Oh we’re not gonna give them this job.”
**Legend** CC = Competency Committee Meeting Field Notes from September 2018 (1) and March 2019 (2) FG = Focus Groups with the Program Director and Competency Committee Chair from September 2018 (1) and March 2019 (2) I = Interviews with Competency Committee Members 1 through 5 in June 2019		

## Appendix B. Quarterly resident self-assessment &reflection template

1. Rotations completed over last 4 blocks
  - ____________________________
  - ____________________________
  - ____________________________
  - ____________________________
2. Upcoming rotations over next 4 blocks
  - ____________________________
  - ____________________________
  - ____________________________
  - ____________________________
3. Other learning activities over last 4 blocks
  - Longitudinal rotations & tasks: EMS, education, admin, collaborator & CANMEDS
    ______________________________________________________________________________
    ______________________________________________________________________________
    ______________________________________________________________________________
    ______________________________________________________________________________
    ______________________________________________________________________________
  - E-learning ( CLR 800, Indigenous Health, Image Sim), courses & conferences
    ______________________________________________________________________________
    ______________________________________________________________________________
    ______________________________________________________________________________
    ______________________________________________________________________________
    ______________________________________________________________________________
  - Academic & professional activities (presentations, EMS tasks, teaching, committees, etc.)
    ______________________________________________________________________________
    ______________________________________________________________________________
    ______________________________________________________________________________
    ______________________________________________________________________________
    ______________________________________________________________________________
  - Scholarly project update
    ______________________________________________________________________________
    ______________________________________________________________________________
    ______________________________________________________________________________
    ______________________________________________________________________________
4. Recognitions (please outline any awards/accomplishments you may have received)
  ______________________________________________________________________________
  ______________________________________________________________________________
  ______________________________________________________________________________
  ______________________________________________________________________________
5. Which of the following do you believe best describes your progress within your current CBME stage (check one) . Current Stage: _____________________________
  **Table:**
  Inactive	Failure to Progress	Not Progressing as Expected	Progressing as Expected	Progress is Accelerated
6. Please provide a short reflective self-appraisal of your progress regarding your learning objectives from last quarter as well as any additional learning and professional development that you feel as has taken place (considering highlighting specific points based on educational activities that you have done, EPA’s achieved and/or review of learning objective goals from last self-assessment/reflection form)
  ______________________________________________________________________________
  ______________________________________________________________________________
  ______________________________________________________________________________
  ______________________________________________________________________________
  ______________________________________________________________________________
  ______________________________________________________________________________
  ______________________________________________________________________________
  ______________________________________________________________________________
7. Please provide a list of 2-3 learning objectives you plan on focusing on during the next 3 blocks and your learning plan to achieve these objectives
  ______________________________________________________________________________
  ______________________________________________________________________________
  ______________________________________________________________________________
  ______________________________________________________________________________
  ______________________________________________________________________________
  ______________________________________________________________________________
  ______________________________________________________________________________
8. Career Plan
  ______________________________________________________________________________
  ______________________________________________________________________________
  ______________________________________________________________________________
  ______________________________________________________________________________
